# TLR9 mediates *S. aureus* killing inside osteoblasts via induction of oxidative stress

**DOI:** 10.1186/s12866-016-0855-8

**Published:** 2016-10-03

**Authors:** Walid Mohamed, Eugen Domann, Trinad Chakraborty, Gopala Mannala, Katrin S. Lips, Christian Heiss, Reinhard Schnettler, Volker Alt

**Affiliations:** 1 Laboratory of Experimental Trauma Surgery Giessen, Justus-Liebig-University Giessen, 35394 Giessen, Germany; 2 Institute of Medical Microbiology, German Centre of Infection Research, Justus-Liebig-University Giessen, 35392 Giessen, Germany; 3 Department of Trauma Surgery Giessen, Justus-Liebig-University Giessen, 35385 Giessen, Germany

**Keywords:** *S. aureus*, CpG-ODN, TLR9, Oxidative stress

## Abstract

**Background:**

*Staphylococcus aureus* is the principle causative pathogen of osteomyelitis and implant-associated bone infections. It is able to invade and to proliferate inside osteoblasts thus avoiding antibiotic therapy and the host immune system. Therefore, development of alternative approaches to stimulate host innate immune responses could be beneficial in prophylaxis against *S. aureus* infection. TLR9 is the intracellular receptor which recognizes unmethylated bacterial CpG-DNA and activates immune cells. Synthetic CpG-motifs containing oligodeoxynucleotide (CpG-ODNs) mimics the stimulatory effect of bacterial DNA.

**Results:**

Osteoblast-like SAOS-2 cells were pretreated with CpG-ODN type-A 2216, type-B 2006, or negative CpG-ODN 2243 (negative control) 4 h before infection with *S. aureus* isolate EDCC 5055 (=DSM 28763). Intracellular bacteria were streaked on BHI plates 4 h and 20 h after infection. ODN2216 as well as ODN2006 but not ODN2243 were able to significantly inhibit the intracellular bacterial growth because about 31 % as well as 43 % of intracellular *S. aureus* could survive the pretreatment of SAOS-2 cells with ODN2216 or ODN2006 respectively 4 h and 20 h post-infection. RT-PCR analysis of cDNAs from SAOS-2 cells showed that pretreatment with ODN2216 or ODN2006 stimulated the expression of TLR9. Pretreatment of SAOS-2 cells with ODN2216 or ODN2006 but not ODN2243 managed to induce reactive oxygen species (ROS) production inside osteoblasts as measured by flow cytometry analysis. Moreover, treating SAOS-2 cells with the antioxidant Diphenyleneiodonium (DPI) obviously reduced *S. aureus* killing ability of TLR9 agonists mediated by oxidative stress.

**Conclusions:**

In this work we demonstrated for the first time that CPG-ODNs have inhibitory effects on *S. aureus* survival inside SAOS-2 osteoblast-like cell line. This effect was attributed to stimulation of TLR9 and subsequent induction of oxidative stress. Pretreatment of infected SAOS-2 cells with ROS inhibitors resulted in the abolishment of the CPG-ODNs killing effects.

## Background

*S. aureus* is the major pathogen causing osteomyelitis and septic arthritis [1] and highly involved in prosthetic joint infections following arthroplasty [2]. Moreover, it was established that *S. aureus* is able to invade and proliferate inside osteoblasts [3, 4] thus avoiding the host extracellular antibacterial weapons.

Innate immunity acts as the first active defense line against infectious pathogens and is responsible for the control of their early invasion and proliferation inside the host [5]. One of the largest and most extensively studied groups of innate immune response receptors are the Toll-like receptors (TLRs). TLRs are evolutionarily conserved transmembrane receptors between insects and vertebrates. They are homologues of the Drosophila Toll protein and have been established to be important for defense against microbial infection [6]. TLRs mainly recognize highly conserved structural motifs known as pathogen-associated microbial patterns (PAMPs), which are exclusively expressed by a wide variety of infectious microorganisms but not by the host. PAMPs include various bacterial components such as lipopolysaccharide (LPS) of Gram-negative bacteria, peptidoglycan (PGN) and lipopeptides of Gram-positive bacteria, as well as flagellin, unmethylated bacterial CPG-DNA and viral double-stranded RNA [7–9].

Stimulation of TLRs by the corresponding PAMPs initiates signaling cascades leading to the activation of transcription factors, such as AP-1 [10], NF-kB [11] and interferon regulatory factors (IRFs) [12]. Previous studies have shown that signaling by TLRs result in a variety of cellular responses including the production of interferons (IFNs), pro-inflammatory cytokines and effector cytokines that direct the adaptive immune response [13, 14].

TLR9 is an intracellular receptor. It recognizes and is stimulated by unmethylated bacterial CpG-DNA and initiates potent cellular and humoral immune responses [13, 15]. Although TLR9 are mainly expressed in immune cells such as dendritic cells, natural killer cells, monocytes and B cells, it was also detected in osteoblasts and its role in modulating the osteoclastogenic activity of osteoblasts was reported [16].

The stimulatory effect of bacterial CpG-DNA is due to the presence of unmethylated CpG dinucleotides in a particular base context named CpG motif [15]. Bacterial genomes and vertebrate genomes are different in the frequency and methylation status of CpG dinucleotides. CpG dinucleotides in bacterial genomes generally occur at a frequency of about 1/16 as predicted by random base utilization and are unmethylated. In contrast, CpG dinucleotides in vertebrate DNA are suppressed to a frequency of only 1/50–60, almost all of which are methylated [17–19]. CpG-ODNs possess similar stimulatory effects as bacterial DNA. The CpG-DNA induces B-*cell* proliferation and activates cells of the myeloid lineage such as dendritic cells [15, 20, 21]. Moreover, it has been recently shown that CpG could mediate peroxide formation in cultured hepatocytes and dendritic cells resulting in an enhanced killing of intracellular *Salmonella* [22, 23].

Multiple studies have demonstrated that unmethylated CpG-ODNs have potent immunostimulatory effects in various immune cell subsets resulting in direct or indirect activation of NK cells, T cells, B cells, monocytes, macrophages and dendritic cells [23–25]. Based on these effects, CpG-ODNs act as immune adjuvants that boost antigen-specific immune responses. These effects are optimized by maintaining close physical contact between the CpG-ODN and the immunogen [26]. Co-administration of CpG-ODN with a variety of vaccines has enhanced humoral and/or cellular immune responses with subsequent stimulation of a protective immunity in various animal models of infectious diseases [27–32]. Moreover, CpG-ODNs have shown impressive effects as a single anti-cancer therapeutic agent in different animal tumor models [33–35] and can increase resistance against acute non-specific polymicrobial sepsis [36].

However, no published data are currently available about the effect of CPG-ODNs on the intracellular *S. aureus* survival in human osteoblasts. We have recently shown that intracellular *S. aureus* survival can be inhibited by antibiotics treatment such as rifampicin. The aim of the current study was to show the impact of CpG-ODNs on the expression of TLR9 in osteoblasts and if they have an inhibitory effect on the intracellular survival of *S. aureus* inside osteoblasts. Moreover, the mechanism of action of CpG-ODNs on the intracellular *S. aureus* will be demonstrated.

## Methods

### Bacteria

Bacterial strain used in this study is *S. aureus* isolate EDCC 5055 (available at DSMZ; number “DSM 28763”) [4, 37]. Bacteria were grown in brain-heart infusion (BHI) broth. In all experiments, fresh cultures of bacteria, prepared from an overnight culture, were used. Briefly, bacteria were grown in Brain Heart Infusion (BHI) at 37 °C, harvested in the exponential growth phase and washed twice with phosphate buffer saline (PBS). The pellet was resuspended in PBS and the bacterial concentration was calibrated by optical absorption. Further dilutions were prepared in PBS to obtain required numbers of bacteria for infection. Hemolytic activity was determined as described [37, 38].

Biofilm formation testing of *S. aureus* EDCC 5055 was based on the ability of the bacteria to form biofilms on polystyrene (PS) plastic, e.g. the development of microcolonies that can be detected macroscopically. Biofilm formation assay was performed as described [39]. Briefly, overnight cultures in TSB were diluted 1:100 into fresh medium and 200 μl of the freshly inoculated medium was dispensed into the wells of a microtiter plate. The inoculated microtiter plate was incubated at 37 °C for 48 h without agitation. The liquid cultures were removed and the wells were rinsed twice thoroughly and vigorously with biofilm buffer (2 mM CaCl_2_/MgCl_2_) to remove unattached cells. Two hundred microliter of biofilm buffer and 20 μl of CV staining solution (0.1 % w/v crystal violet in water) were added to detect biofilms by staining. The plates were incubated for 15 min at room temperature and then rinsed with biofilm buffer to remove residual staining. Bacteria able to form biofilms coated the inner surface of the wells and the microcolonies were visible in purple colour.

### ODNs

ODN2216 (InvivoGen) is an A-class CpG ODN with a preference for human TLR9 characterized by a phosphodiester central CpG-containing palindromic motif and a phosphorothioate 3’ poly-G string. A-class CpG ODN mainly stimulates and induces secretion of IFN-α from plasmacytoid dendritic cells [40].

ODN2006 (InvivoGen) is a CpG ODN class B specific for human TLR9. It contains a full phosphorothioate backbone with one or more CpG dinucleotides. B-class CpG ODN is a strong stimulator for B-cells [41].

ODN2243 (InvivoGen) is designed as a negative control. ODN2243 contains GpC dinucleotides instead of CpGs and does not induce TLR9 activity [42].

### In vitro invasion assay of SAOS-2 cells

SAOS-2 osteoblast-like cell line was grown in Gibco Minimum Essential Medium (MEM) (*Life technologies*) supplemented with 10 % fetal calf serum (FCS) and 1 % non-essential amino acids. For infection assay, SAOS-2 cells were cultured to a semi confluent layer (2x10^5^ cells/ml) in 24-well plates. Cells were treated with ODN2216, ODN2006, or ODN2243 (250 nM) (InvivoGen) 4 h prior to infection with *S. aureus*. 10 μM diphenyleneiodonium chloride (DPI) (Sigma-Aldrich) was added to ODN-pretreated cells 1 h prior to infection in order to confirm the role of oxidative stress in *S. aureus* intracellular survival. Bacterial cultures were incubated overnight. Following 1:10 fresh media dilution, bacterial cultures were grown for 2 h and diluted to an OD of 0.1 with MEM medium. Bacteria were added at a multiplicity of infection (MOI) of 30 per well. After incubation for 30 min, supernatant was discarded and infected cells were washed twice with 1x PBS. MEM was replaced by medium supplemented with 30 μg/ml gentamicin to kill only the remaining extracellular bacteria without affecting the intracellular bacteria [43]. Cells were then incubated at 37 °C for 2, 4, 6 and 24 h. The supernatant fluids were discarded and the cells were washed three times with 1x PBS and lysed with 0.2 % Triton X-100 in sterile cold distilled water for 20 min after this cells were thoroughly mixed to achieve complete lysis. The lysates were diluted 10 times in 1x PBS and plated onto BHI agar plates using the Auto plate 3000 spiral plating system (Spiral Biotech, USA). After 24 h of incubation at 37 °C, the number of bacterial colonies were counted and the total colony forming units (cfu) were determined. In order to determine the expression of TLR9 and induction of oxidative stress in SAOS-2 cells, cells were treated with ODN2216, ODN2006, ODN2243 (250 nM) or left untreated 4 h prior to lysis for RNA isolation or collection for flow cytometry analysis.

### RNA isolation

SAOS-2 cells from three wells of a six well tissue culture plaque were lysed using RLT lysis buffer (Qiagen) after aspiration of the MEM medium. Total RNA was isolated using the RNeasy Mini Kit and the RNase free DNase I set (Qiagen) following the manufacturers protocol. The RNA was recovered in RNase free water, heat denatured for 10 min. at 65 °C; quantified with the NanoDrop® ND-1000 UV–vis Spectrophotometer (NanoDrop Technologies) and a quality profile with the Agilent 2100 bioanalyzer (Agilent Technologies) was made.

### Real time RT-PCR

First-strand cDNA was synthesized from 500 ng of purified RNA using SuperScriptII (Invitrogen) and a mixture of T21 and random nonamer primers (Metabion) following the instructions for the reverse transcription reaction recommended for the QuantiTect SYBR Green PCR Kit (Qiagen). Real-time quantitative PCR was performed on an ABI Prism 7700 real time cycler using human TLR9 primers (Qiagen). Human ACTB primer pair (Qiagen) was used as a positive control. The PCR products underwent agarose gel electrophoresis.

### Measurement of oxidative stress by flow cytometry

SAOS-2 cells pretreated with ODNs were detached from cell culture plates. To detect ROS formation, we stained the cells for 30 min with the fluorescent reporter dye 3′-(p-hydroxyphenyl) fluorescein (HPF, Invitrogen) at a concentration of 5 μM at room temperature. Cells were washed twice with PBS to remove excess HPF and suspended in PBS supplemented with 1 % FCS. Flow cytometry was performed using a FACS Calibur flow cytometer and further analysed with CELL Quest software (Becton Dickinson).

### Statistical analysis

Data are representative of at least three independent experiments. Significance of the represented data was calculated using unpaired Student’s *t* test. Data are expressed as mean ± standard errors.

## Results

### CpG-ODNs control survival of *S. aureus* inside osteoblasts

**Fig. 1:**
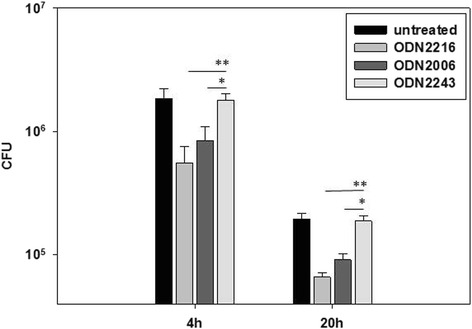
CpG-ODNs inhibit intracellular growth of *S. aureus* in SAOS-2 osteoblast-like cell line. SAOS-2 cells were pretreated with 250 nM of ODN2216, ODN2006, or ODN2243 4 h before infection or left untreated. Bacteria infected osteoblasts at a MOI of 30 for 30 min after which gentamicin (30 μg/ml) was added and intracellular bacterial growth was monitored at 4 h and 20 h post-infection (**P < 0, 05; *P < 0, 10)

*S. aureus* EDCC5055 has been previously shown to successfully invade and proliferate inside SAOS-2 cells where bacteria could successfully survive inside osteoblasts during the first 6 h after infection. However, intracellular bacterial survival was dramatically reduced 20 h after infection in comparison to 4 h post-infection due to induction of apoptosis in SAOS-2 cells thus exposing the intracellular bacteria to the action of the extracellular gentamicin [4]. In this study, pretreatment of SAOS-2 cells with ODN2216 or ODN2006 could clearly inhibit intracellular survival of *S. aureus* as the number of intracellular bacteria was significantly reduced 4 h post infection. This inhibitory effect could be also observed 20 h post infection. Pretreatment of SAOS-2 cells with ODN2243 did not show any obvious reduction in intracellular survival of *S. aureus* either 4 h or 20 h post-infection in comparison to untreated cells (Fig. 1).

### CpG-ODNs induce TLR9 expression in SAOS-2 cells

**Fig. 2:**
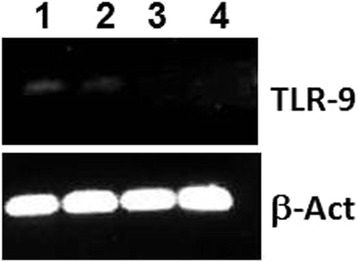
CpG-ODNs induce the expression of TLR9 in SAOS-2 cells. SAOS-2 cells were treated for 4 h with 250 nM of ODN2216 (1), ODN2006 (2), ODN2243 (3) or left untreated (4). cDNA was synthetized from isolated SAOS-2 RNA and underwent real-time quantitative PCR using human TLR9 or ACTB primers. PCR products were analyzed on 1 % agarose gel

TLR9 is normally expressed by various immune cells such as dendritic cells, B lymphocytes, monocytes and natural killer (NK) cells but could also be expressed by epithelial non-phagocytic cells such as osteoblasts [16]. Using quantitative RT-PCR for cDNAs generated from RNAs of SAOS-2 cells which were pretreated with different CpG-ODNs, we could show that expression of TLR9 in SAOS-2 osteoblast-like cells was upregulated following treatment with ODN2216 and ODN2006 while pretreatment of SAOS-2 cells with ODN2243 was not able to stimulate TLR9 expression as shown by agarose gel electrophoresis (Fig. 2). β-Actin (ACTB) showed the same expression quantity under all conditions.

### CpG-ODNs enhance TLR9-mediated oxidative stress in SAOS-2 cells

**Fig. 3:**
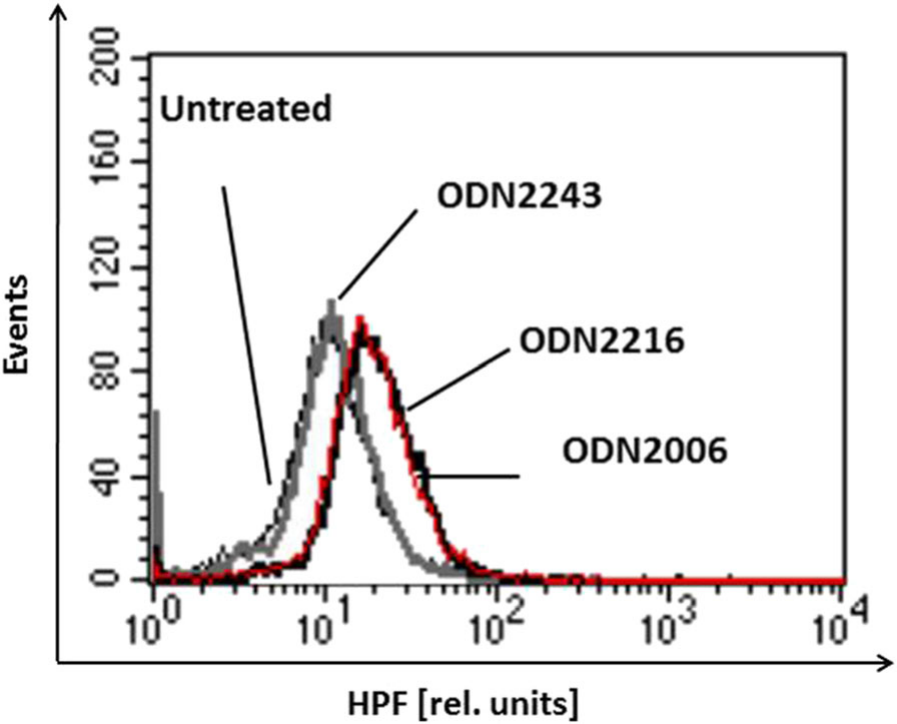
CpG-ODNs stimulate production of ROS by SAOS-2 cells. SAOS-2 cells were treated for 4 h with 250 nM of ODN2216, ODN2006, ODN2243 (Neg. ODN) or left untreated. Cells were then stained with the ROS indicator, HPF. Binding of HPF to SAOS-2 cells was analysed by flow cytometry

**Fig. 4:**
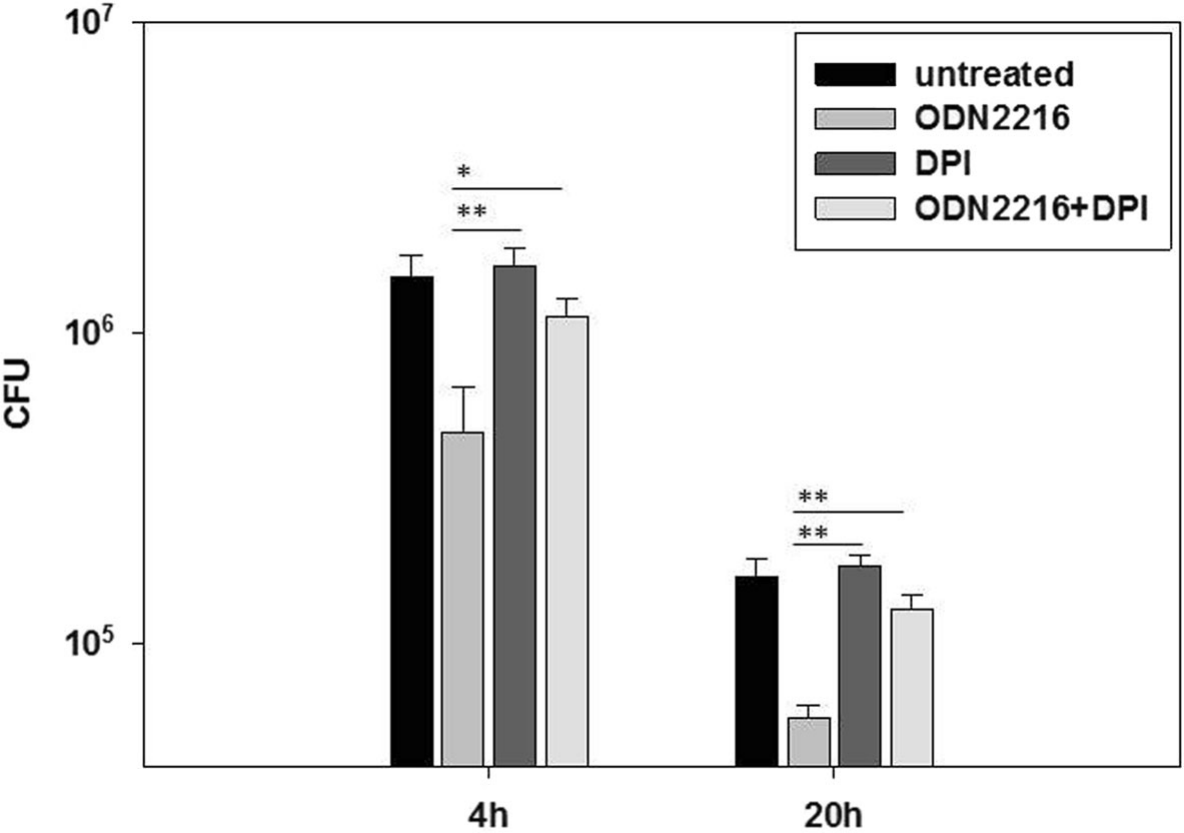
The antioxidant DPI reduces the intracellular growth inhibitory effect of ODNs on *S. aureus*. SAOS-2 cells were pretreated with 250 nM of ODN2216 4 h and/or with 10 μM of DPI 1 h before infection or left untreated. Bacteria infected osteoblasts as described in Fig. 1 (**P < 0, 05; *P < 0, 10)

In order to explain the mechanism of intracellular bacterial killing in SAOS-2 cells following treatment with ODN2216 and ODN2006, induction of oxidative stress inside osteoblasts was examined after treatment with different ODNs. Pretreatment of SAOS-2 cells with ODN2216 or ODN2006 managed to induce ROS production inside osteoblasts as shown by flow cytometry through shift in fluorescence of HPF-stained SAOS-2 cells (Fig. 3) while pretreatment with ODN2243 as well as ODN-untreated cells did not show any enhancement of ROS production by SAOS-2 cells. Failure of ODN2243 to induce ROS production inside osteoblasts confirms the involvement of TLR9 stimulation in oxidative stress induction inside SAOS-2 cells. Diphenyleneiodonium (DPI) has frequently been used to inhibit ROS production mediated by flavoenzymes, particularly NADPH oxidase [44]. Treating SAOS-2 cells with the antioxidant DPI obviously reduced *S. aureus* killing ability of the TLR9 agonist, CpG-ODN2216, because survival of intracellular *S. aureus* has significantly increased 4 h and 20 h after infection in SAOS-2 cells pretreated with both ODN2216 and DPI in comparison to treatment with ODN2216 alone indicating that intracellular *S. aureus* was killed throughTLR9-mediated oxidative stress (Fig. 4).

Treatment of SAOS-2 cells with only DPI without CpG-ODN did not affect the intracellular survival of *S. aureus* as bacteria could grow at a level equal to that shown in untreated SAOS-2 cells.

## Discussion

*S. aureus* infections are considered not only the most serious cause of implant, prosthetic device and intravascular line infection but also the most common cause of hospitalization for surgical drainage of pus in children or treatment of bacteremia in elderly [45–47]. Elevated levels of mortality, morbidity, and costs resulting from invasive *S. aureus* infections despite the introduction of several new antibiotics encouraged investigators to develop effective alternative interventional strategies to control these infections [47, 48]. Passive immunotherapy with anti *S. aureus* glucosaminidase monoclonal antibody has recently shown promising treatment potential in mouse model of implant-associated osteomyelitis [49].

During infection bacterial DNA is released and exposes host cells that express TLR9 to the unmethylated CpG motifs, thus stimulating a protective immune response against the invading pathogen [13, 15]. The immunostimulatory activity of bacterial DNA is mimicked by CpG-ODNs [50]. After internalization by target cells, CpG-ODNs reach the late endosomal/lysosomal compartment where they signal by interacting with TLR9 [51]. TLR9 knockout mice do not respond to stimulation by CpG-ODNs [13].

Osteoblasts have been recently shown to express TLR9 receptors that are able to transmit cellular signaling and induce osteoblasts osteoclstogenic activity [16]. *S. aureus* can invade and proliferate inside osteoblasts [3, 4] thus remains protected from extracellular immune responses such as antibodies, cationic peptides and recognition by phagocytic cells in addition to antibiotics. This evasion strategy plays a crucial role in recurrence and persistence of *S. aureus* infection. Therefore, stimulation of host innate immune responses could be an efficient approach in prophylaxis against *S. aureus* infection.

In this study we have shown for the first time that synthetic CpG-ODNs could significantly inhibit *S. aureus* intracellular survival inside SAOS-2 cells, an effect which negative CpG-ODN was not able to show. CpG-ODNs could induce expression of TLR9 in SAOS-2 cells indicating that intracellular bacterial killing effect of CpG-ODNs could be mediated by TLR9.

During the initial stages of infection by *S. aureus*, phagocytes including neutrophils and macrophages are recruited to the site of infection and engulf the pathogen with subsequent killing in the phagolysosome by production of lysosomal enzymes and ROS [52]. In fact, production of ROS is a common antibacterial mechanism elicited by professional antigen presenting cells in order to kill engulfed bacteria in the intracellular compartments [53]. Patients who are neutropenic or have congenital or acquired defects in polymorphonuclear neutrophils (PMNs) function are more susceptible to *S. aureus* infection [54]. The NADH-dependent phagocytic oxidase produces superoxide, which upon dismutation forms hydrogen peroxide. Both superoxide anions and hydrogen peroxide can damage a variety of biomolecules including DNA, proteins, and lipids. Moreover, these species can destroy iron-sulfur clusters with subsequent release of iron which can undergo a Fenton reaction with hydrogen peroxide to yield hydroxyl radical which damages any biological molecule in a diffusion limited manner [55]. Anti-oxidant enzymes such as superoxide dismutase and catalase form part of a defense mechanism that helps *S. aureus* to protect itself from oxygen toxicity [44].

We have previously shown that intracellular *S. aureus* were surrounded by phagosomal/lysosomal membranes inside SAOS-2 cells [4], indicating that intracellular *S. aureus* could be exposed to the action of ROS inside osteoblasts. Our flow cytometry analysis revealed that CpG-ODNs stimulated ROS production by SAOS-2 cells. ROS were able to inhibit intracellular survival of *S. aureus* inside osteoblasts because pretreatment of SAOS-2 cells with the antioxidant, DPI could significantly restore the intracellular growth of *S. aureus*. Our study, in combination with our own previous work and the results of other groups on implant infections, shed light on a complex interplay of *S. aureus* at the interface of implant surface and host tissue. The pathogen, able to form a biofilm on implant surfaces, induces potentially an implant-associated infection and is capable of occasionally releasing planktonic cells thus causing bacteremia or even sepsis. Apart from its capacity to form biofilms, *S. aureus* is able to invade the surrounding tissue which constitutes a niche to escape the attack of the immune system and circulating antibiotics. CpG-ODNs, as shown for the first time in this study, exhibit a new weapon to prevent the nidation of *S. aureus* in the surrounding tissue and osteoblasts. A pre-clinical trial in goats showed promising effects of CpG-ODN against mastitis caused by *E. coli* infection. It induced the expression of TLR9 mRNA in mammary tissue and reduced bacterial count in milk [56].

**Fig. 5:**
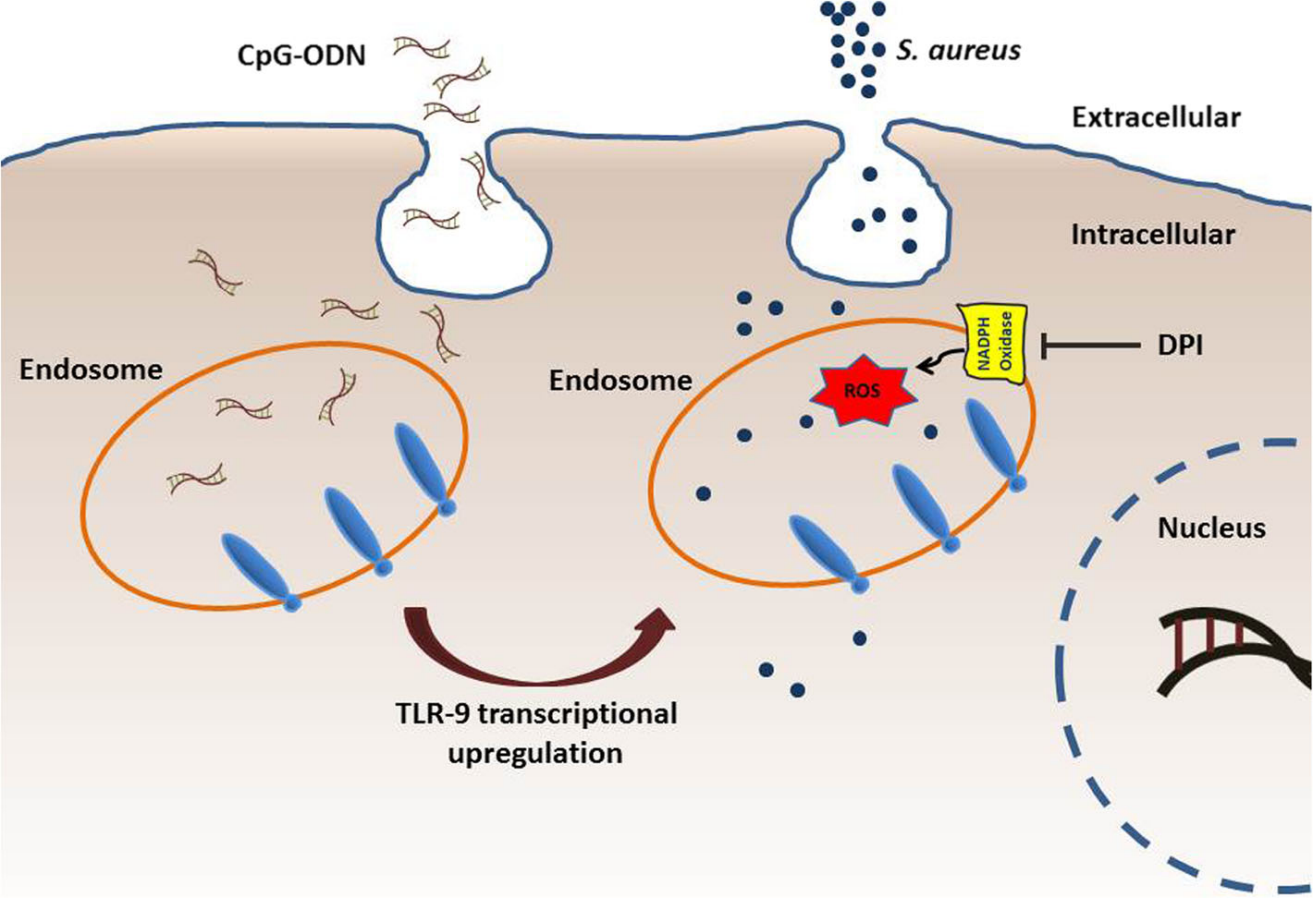
A schematic representation for the pretreatment of SAOS-2 cells by CpG-ODNs prior to infection with *S. aureus*. CpG-ODNs were internalized into the endosomal vesicles that contain TLR9. Recognition of CpG-ODNs by TLR9 upregulates the expression of TLR9 which in turn mediates formation of ROS by NADPH oxidase leading to killing the intracellular *S. aureus* by oxidative stress . Pretreating SAOS-2 cells with Diphenyleneiodonium (DPI), a potent NADPH Oxidase inhibitor, restored the intracellular survival of *S. aureus*

In addition, ongoing clinical studies indicate that CpG-ODNs are safe and well-tolerated when administered as a monotherapy or as adjuvants to humans [57, 58]. In this work, it was revealed that a single administration of CPG-ODNs can induce oxidative stress through stimulation of TLR9 in epithelial non-phagocytic cells (Fig. 5) thus mounting innate immunity against invading infectious pathogens.

## Conclusions

In this study, CpG-ODNs could show promising effects against intracellular *S. aureus*. They managed to induce the expression of TLR9 in SAOS-2 cells with subsequent production of ROS leading to killing of intracellular *S. aureus*. Pretreatment of the infected SAOS-2 cells with DPI could restore the intracellular survival of *S. aureus*. Therefore, CpG-ODNs are promising new tools to support interruption of the interplay of *S. aureus* at the interface of implant devices and surrounding tissue.
